# Catalytic Oxidation of Lignins into the Aromatic Aldehydes: General Process Trends and Development Prospects

**DOI:** 10.3390/ijms18112421

**Published:** 2017-11-15

**Authors:** Valery E. Tarabanko, Nikolay Tarabanko

**Affiliations:** Institute of Chemistry and Chemical Technology SB RAS, Federal Research Center “Krasnoyarsk Science Center SB RAS”, Akademgorodok 50/24, Krasnoyarsk 660036, Russia; chem@icct.ru

**Keywords:** lignin, lignosulfonates, wood, vanillin, syringaldehyde, retroaldol reaction, mechanism, technology

## Abstract

This review discusses principal patterns that govern the processes of lignins’ catalytic oxidation into vanillin (3-methoxy-4-hydroxybenzaldehyde) and syringaldehyde (3,5-dimethoxy-4-hydroxybenzaldehyde). It examines the influence of lignin and oxidant nature, temperature, mass transfer, and of other factors on the yield of the aldehydes and the process selectivity. The review reveals that properly organized processes of catalytic oxidation of various lignins are only insignificantly (10–15%) inferior to oxidation by nitrobenzene in terms of yield and selectivity in vanillin and syringaldehyde. Very high consumption of oxygen (and consequentially, of alkali) in the process—over 10 mol per mol of obtained vanillin—is highlighted as an unresolved and unexplored problem: scientific literature reveals almost no studies devoted to the possibilities of decreasing the consumption of oxygen and alkali. Different hypotheses about the mechanism of lignin oxidation into the aromatic aldehydes are discussed, and the mechanism comprising the steps of single-electron oxidation of phenolate anions, and ending with retroaldol reaction of a substituted coniferyl aldehyde was pointed out as the most convincing one. The possibility and development prospects of single-stage oxidative processing of wood into the aromatic aldehydes and cellulose are analyzed.

## 1. Introduction

Traditionally utilized industrial technologies of wood chemical processing provide a limited assortment of primary products—cellulose, other carbohydrates—and substances derived from them. The subject of processing lignin into valuable chemicals has been attracting research for over 100 years, and presently remains actively discussed [1,2,3,4,5,6,7].

Among promising approaches to disposal of lignins, their processing into the aromatic aldehydes—vanillin (4-hydroxy-3-methoxybenzaldehyde) and syringaldehyde (4-hydroxy-3,5-dimethoxybenzaldehyde)—can be highlighted [1,2,3,4,5,6,7,8,9,10]. These substances are important in pharmaceutical, food, and fragrance industries [11,12]. Vanillin is used for the production of papaverine, ftivazide, and l-DOPA [13]. Active research is devoted to the vanilloid class of drugs, e.g., capsaicin—a component of cayenne pepper and the basis of medical topical formulations [13,14,15]. Syringaldehyde can be used for the synthesis of trimethoxybenzaldehyde, trimethoprim and other pharmaceuticals [16]. Syringaldehyde can also be transformed into substituted anthraquinones—the catalysts for improving the processes of alkaline delignification [17]. *Para*-hydroxybenzaldehyde finds use in polymer synthesis and other branches of the chemical industry. Examples of polymer synthesis from lignin-derived monomers are polyferulic acid, polydihydroferulic acid, and copolymers of ferulic or coumaryl acid with ethylene glycol—more environmentally benign alternatives to poly(ethylene terephthalate) [18].

Vanillin production by oxidation of lignosulfonates (the byproduct of sulfite wood pulping) dominated the world market from the 1950’s to the 1970’s, and is still in use in Scandinavia. Presently, vanillin is primarily produced from guaiacol and glyoxylic acid, attaining around 15,000 tonnes per year at the price 6–15 USD/kg. Developing more efficient methods of vanillin production from lignins can lead to the price decrease and emergence of novel large-scale technologies of its consumption, like production of new polymers [19,20,21].

A considerable amount of experimental data in the field of lignins processing has been accumulated and there is a number of modern reviews, this also includes the subject of catalytic oxidation [2,3,4,5,6,7]. However, the lately published reviews barely look into the principal patterns that govern the processes in question: how the aldehyde yield and selectivity are influenced by the nature of lignin and oxidant, temperature, mass transfer, etc. The prospects for developing the industry of the aromatic aldehydes production by oxidation of lignins are not discussed either.

The purpose of this review is the analysis of the modern literature data on catalytic oxidation of lignins by oxygen into vanillin and syringaldehyde, from basic patterns and mechanisms to the prospects of industrial implementation.

## 2. Lignin Structure

Lignin is one of the principal components of plant biomass. Its content in wood attains 30 wt. %. Considerable amounts of technical lignins (Kraft lignin, lignosulfonates, hydrolysis lignin, etc.; more about their differences from native lignin later in this Section) are generated after chemical processing of wood by the pulping, paper, and hydrolysis industries.

**Scheme 1:**
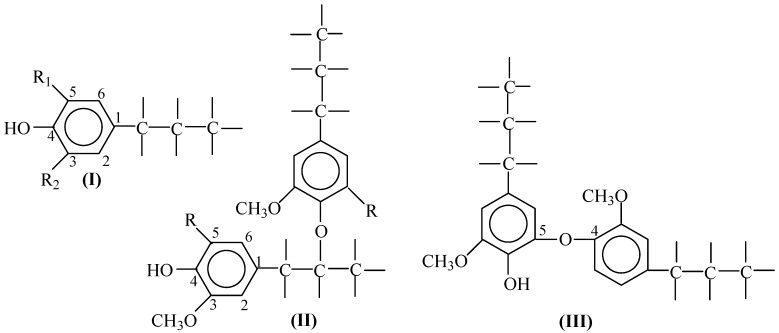
Lignin structural unit, and the principal types of linkage between the units.

The plant cell wall is an intricate biochemical complex formed primarily by cellulose, hemicelluloses, and lignin [1,22]. The structure of lignin (Scheme 1) is comprised of phenylpropane units (PPU, (**I**)).

Softwood lignin has simple composition mostly consisting of guaiacylpropane structural units (R_1_ = H, R_2_ = OMe). Hardwood lignin contains, in addition to those, syringylpropane units (R_1_ = R_2_ = OMe). Besides that, grass lignin also includes *p*-hydroxyphenylpropane units (R_1_ = R_2_ = H). Softwood and hardwood contain 28–30% and 18–24% of lignin, respectively [1,22]. Grassy plants contain relatively little lignin (5–15%), and its oxidation leads to the most complex aldehyde mixture—vanillin, syringaldehyde, and *p*-hydroxybenzaldehyde. For these reasons, oxidation of grass lignins is not discussed in detail in this review.

The principal functional groups in lignin are methoxyl substituents at the benzene ring, alcoholic and phenolic hydroxyls, carbonyl (both aldehyde and ketone) and carboxylic groups. The content of the methoxyl groups is 15–16 wt. % in softwood lignins and 17–21% in hardwood lignins. The elemental composition of lignin is 61–64 wt. % carbon, 5–6% hydrogen, balanced with oxygen [22].

Among the numerous types of linkage between PPUs, it is worth pointing out the β–O–4 bonds (**II**) as the type that may be dominant in native lignins. Additionally, the 5–O–4 (**III**), 5–5 and 5–β bonds are created while pulping wood, processing and isolating the lignins of guaiacyl structure. These three bond types are the result of condensation reactions involving the benzene ring 5th position [1,22]. As the first approximation, the described bond types may suffice to explain the general patterns of lignin transformation in oxidation processes. Hardwood lignins have abundant syringyl PPUs that already have their aromatic fifth position substituted, and are unable to undergo condensation involving this position like guaiacyl PPUs in softwood lignins do. However, syringyl PPUs still can undergo condensation with formation of syringaresinol structures [22].

The structural units with free (non-etheric) phenolic functional groups have high reactivity, particularly in terms of oxidation in alkaline media. The carbohydrate components of wood, especially cellulose, are considerably more stable in these conditions. Oxidation of lignins in alkaline media leads first of all to destruction of the propane chains, while the aromatic rings retain their aromatic structure. Oxidation of lignins in acidic aqueous solutions leads primarily to the products of the benzene ring destruction [1].

## 3. Oxidation of Lignins into the Aromatic Aldehydes by Nitrobenzene

Oxidation of lignins by nitrobenzene—or nitrobenzene oxidation (NBO)—is a long-known [23,24,25] and highly selective process; its results qualitatively—and even quantitatively—were used to devise the structure of lignins [1,8,22,23,24]. The use of nitrobenzene for oxidizing isoeugenol into vanillin was patented in 1927 [25]. NBO of lignins was actively developed in the works of Hibbert, Leopold, and other authors [23,24,25,26,27,28,29,30,31,32,33,34,35,36,37]; new inquiries into the process are still being made [27,28] (Table 1). The process takes place in alkaline media at 160–180 °C lasting 2–4 h [23]. NBO causes oxidative lignin destruction with the α–β bond cleavage, leading to formation of the aromatic aldehydes as the principal products, together with their corresponding carboxylic acids, and a small portion of the aceto-derivatives of the main products—acetovanillone (4-hydroxy-3-methoxyacetophenone) and acetosyringone (4-hydroxy-3,5-dimethoxyacetophenone). The yields of these products characterize the quantities (and their ratios) of non-substituted guaiacyl and syringyl units in the macromolecules of the initial lignin.

The aldehyde yield depends on the lignin source, the conditions of its isolation or preprocessing, and the conditions of lignin oxidation. The highest aromatic aldehyde yields are attained in the nitrobenzene oxidation of native (in the original timber) hardwood lignins, up to 40–50 wt. %; even higher figures (63–64 wt. %) were obtained by using the catalytic systems nitrobenzene-phenanthroline and nitrobenzene-anthraquinone [31,38]. Vanillin yields from softwood lignins do not exceed 25–30 wt. %.

Insignificantly altered lignin, e.g., the lignin of milled pine wood, yields practically the same vanillin amount as the native one (23.4 wt. % and 24.8 wt. % respectively) [33,35]. A small vanillin yield decrease (from 27 to 23–24 wt. %) [34] or even no decrease at all [33] are caused by switching from native softwood lignin to its brown rotted form or enzymatic lignin. A more considerable decrease in the yield of the aldehydes is observed between aspen wood and its enzymatic lignin (from 44 to 34 wt. %) [39].

Oxidation of isolated technical lignins yields 5–16 wt. % of the aromatic aldehydes. Among the technical lignins outlined in Table 1, the aromatic aldehydes yield decreases along the list of the wood processing techniques: sulfite > Kraft > soda-anthraquinone > soda > hydrolysis.

One reason behind these yield patterns is that the syringyl structural units are more stable than the guaiacyl ones because the former cannot undergo condensation (1) involving the unsubstituted fifth carbon atom in the benzene ring [40,41]: here R = CH_3_O or H. The competition between Reactions (1) and (2) explains the differences in the aldehyde yields between hardwood and softwood oxidation, as well as (to some extent) the decrease of the aldehyde yield among technical lignins [40,41,42]. The lignin condensation extent is increasingly affected along the list of technical lignins mentioned above by the harsh conditions of lignin treatment in strong alkaline or acid media together with high temperature; therefore, the vanillin yield decreases along this list. Higher temperature of Kraft cooking compared to the sulfite process (180 and 130 °C, correspondingly [22]) may cause deeper condensation of Kraft lignin.

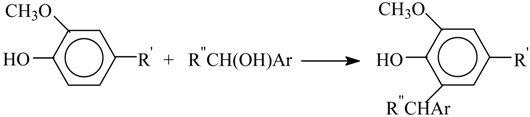
(1)

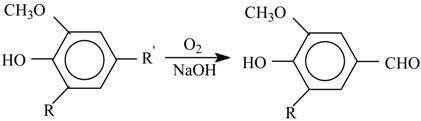
(2)

As a final remark for this Section, it should be emphasized that the quantitative results of lignin oxidation by nitrobenzene can be used as the reference value of the highest practically attainable vanillin and syringaldehyde yield in the catalytic oxidation of the corresponding ligneous feedstock by oxygen.

## 4. Catalytic Oxidation of Lignins by Oxygen

In the first half of the 20th century, it was discovered that the processes of lignosulfonate oxidation into vanillin by oxygen are catalyzed by oxides of copper, manganese, cobalt, and silver [24], and these catalysts exhibit similar effectiveness. For example, catalyzing the oxidation of native spruce lignin by the oxides of either copper or manganese yields practically the same vanillin amounts (18–19 wt. %, Table 2). In alkaline media, the hydroxides of the aforementioned metals—Cu(II), Ag(I), Mn(IV), Co(III)—have relatively low redox potential (below 0.35 V), among which copper has the lowest. As will later be discussed in the Section about the process mechanism, catalysts for obtaining the aldehydes indeed should have low redox potential.

Using catalysts increases the aromatic aldehydes yield in oxidation of lignins by oxygen by a factor of 1.5–2 [1,22,24,42,43,44,45,46,47,48,49]. Comparing the yields of the main products in the nitrobenzene oxidation and the catalytic oxygen-consuming processes reveals that typically the latter are not much worse than the NBO. Across different lignin types (Table 2), the average value of the ratios between the aldehyde yield in nitrobenzene and catalytic processes conducted under similar conditions (160–180 °C, 80–100 g/L NaOH) is 0.87 ± 0.07 (arithmetic mean error). Therefore, properly organized catalytic processes of various lignins oxidation are at most 10–15% inferior to nitrobenzene oxidation in terms of vanillin and syringaldehyde yields.

Naturally, the lignin origin affects the aldehydes yield, and the trends observed for the catalytic oxidation are similar to those for the NBO. The relatively high aldehydes yield from the hydrolysis lignin of poplar in the presence of copper- and iron-containing hydroxides should be pointed out (up to 15 wt. %) [55]. This is quite a high figure for hydrolysis lignins, and it can be explained by an insignificant extent of intermolecular condensation in the lignin dissolved under mild percolation timber hydrolysis conditions (0.07 wt. % sulfuric acid, 210–220 °C). The soluble hydrolysis lignin of bagasse also yields up to 15 wt. % of vanillin and syringaldehyde, together with 3–4% of their aceto-derivatives [55]. There is also an earlier report about obtaining 8 wt. % of vanillin from the “caramel” (the precipitate from prehydrolysis liquor) of pine wood processing (Table 1) [36]. Therefore, despite the fact that practically no aldehydes can be obtained from the lignins of harsh wood hydrolysis (Klason lignin), there exist certain hydrolysis conditions that leave lignin suitable for oxidation with high yields of the aromatic aldehydes.

In oxidation of the enzymatic hydrolysis lignin from steam-exploded corn cobs, at 120 °C and 5 bar oxygen partial pressure (20 bar total pressure) in an alkaline aqueous media, catalyzed by the perovskite-structured oxides LaFe_1−*x*_Cu*_x_*O_3_ (*x* = 0, 0.1, 0.2), the highest aldehyde yields (2.4% *p*-hydroxybenzaldehyde, 4.6% vanillin, 12% syringaldehyde, based on the lignin mass) were obtained with the catalyst of the highest copper content (*x* = 0.2) [56]. These yields are 1.5–2 times greater than in the non-catalytic reaction; adding the chlorides of Fe(III) or La(III) do not lead to the aldehydes yield increase in these conditions. The data on the effectiveness of the NBO or the catalytic activity of CuCl_2_ in the cited process are lacking [56].

The maximum aldehydes yield in oxidation of the lignin from Dedine fast hydrolysis of bagasse with the Pd/γ-Al_2_O_3_ catalyst attains 15–18 wt. % at 140 °C [57]. The yield difference between the catalytic and non-catalytic variants of the described process is unique, 10–20-fold. Unfortunately, this study offers no comparison of this highly efficient palladium catalyst to copper oxide; data on oxidation by nitrobenzene are also lacking [57]. The latter circumstance makes it impossible to rate the palladium catalyst versus the maximum attainable yield—the figure that tends to greatly vary depending on the conditions of the ligneous material hydrolysis, as previously established (Table 1 and Table 2).

Research into the oxidation of lignins in weakly acidic organic solvent media [58,59,60,61] is worth a special mention. The clear advantage of the processes in acidic media is the lack of alkali consumption, but in practice this may be compensated by the loss of organic solvents to oxidation. These works focus on the oxidation of Kraft and organosolv lignins in methanol and acetic acid. In such media, oxidation of undissociated phenols takes place, as opposed to more reactive phenolate ions in alkaline aqueous media. For this reason, the processes in organic solvents attain the highest efficiency at higher temperatures (170–210 °C and higher) than necessary for the alkaline oxidation. In organic solvents, the yield of vanillin together with methyl vanillate attains 4–7 wt. % based on the lignin, whereas the nitrobenzene process yields 10.6%. Among the studied catalysts (phosphomolybdic acid, chlorides of copper, iron, and cobalt, sulfate of copper), CuSO_4_ and CoCl_2_ are the most effective, but the product yield is at most 30% higher in comparison to the non-catalytic process [59].

With the Co–Mn–Zr–Br catalyst, a similar process proceeds starting with a lower temperature of 140 °C; at 190 °C, up to 10.6 wt. % of vanillin, syringaldehyde, and their corresponding acids was obtained from a mixed organosolv lignin of various hardwoods. This is twice as much compared to the non-catalyzed process [62].

The use of catalysts in the processes of lignin oxidation by hydrogen peroxide [63], as well as lignin oxidation by electrochemical [64] and photochemical [65] methods do not result in high vanillin yield. For the same reason, the use of ionic liquids for the aromatic aldehydes synthesis does not look promising [66].

Next, some trends observed in the catalytic processes will be discussed to gain some insight into why the high results outlined in Table 2 can be attained.

## 5. The Effects of Diffusion in the Processes of Lignin Oxidation

Even in case of solid wood, the catalytic oxidation of lignin by oxygen proceeds considerably faster than with nitrobenzene (0.5–1 and 2–4 h respectively), and is 3–4 times faster when oxidizing lignosulfonates [24,33,43]. Naturally, a question arises: what is the role of oxygen diffusion in the processes of oxidizing lignins, and how significant is this role? 

In the works devoted to the kinetics of powdered aspen and pine wood oxidation in a shaking batch reactor with high mass exchange intensity (shaking rate 4 s^−1^) [44,67,68], it was found that chemical-controlled process kinetics can be implemented at temperatures no greater than 160 °C [67]. At 160 °C the process enters the combined control mode which is evidenced by the decreasing apparent activation energy as the temperature increases. This means that the effects of limitation by diffusion can be expected to play a considerable role in other processes of lignin oxidation in the temperature range around 160 °C.

**Figure 1:**
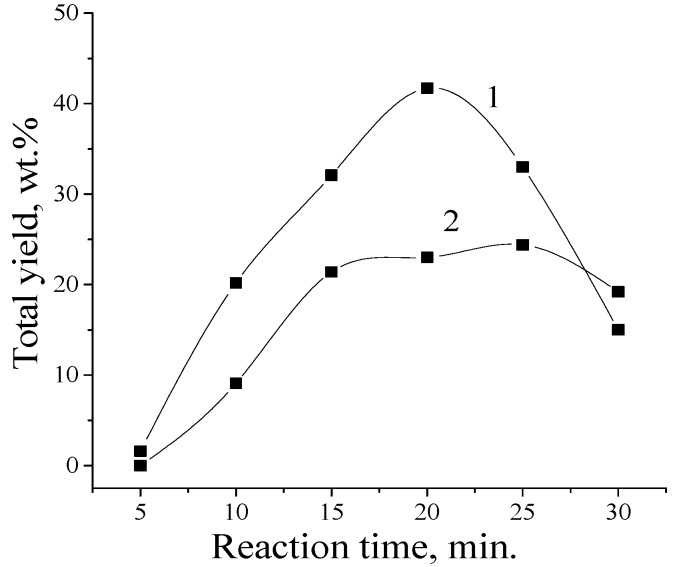
Total yield of the aromatic aldehydes versus process duration in the catalytic oxidation of birch wood by oxygen at 170 °C [51]. (1) 50 g/L loading of wood powder, (2) 100 g/L loading.

Indeed, significant influence of diffusion on the selectivity of powdered birch wood catalytic oxidation was discovered [51]. Decreasing the wood content in the reaction mass from 100 to 50 g/L led to almost two-fold greater yield of the aldehydes—43 wt. % combined based on the lignin, 30% syringaldehyde (Figure 1) [51].

**Scheme 2:**
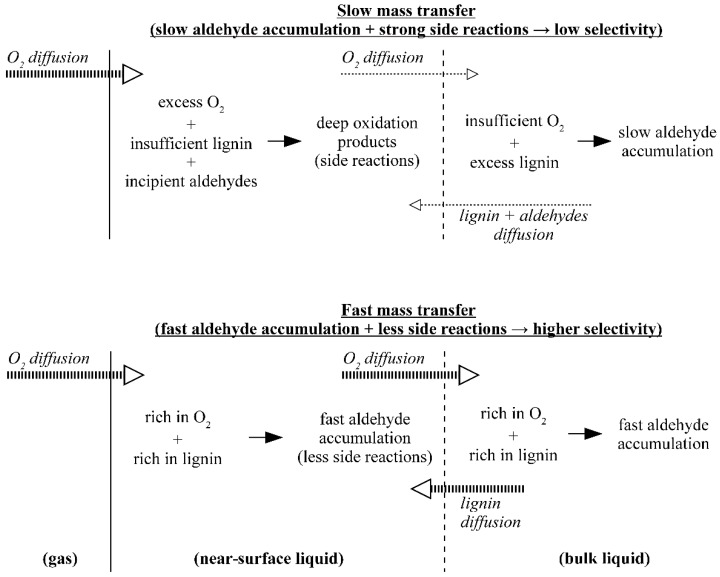
The influence of mass transfer intensity on the selectivity of oxidation of lignins by oxygen. For presentation simplicity, this schematic only includes the factors that have the main effect on the selectivity. Thin solid arrows indicate chemical transformations, thin dashed arrows indicate slow mass transfer, thick dashed arrows indicate fast mass transfer.

In many instances of oxidizing lignosulfonates or other ligneous materials, an increase in the rate of oxygen consumption is observed when the content of the lignin-containing material decreases in the reactor, and this can be explained by diffusion limitations of the process, and by decreased reaction mass viscosity [42,43,51,69,70]. Something less straightforward but quite doubtless is the influence of mass transfer rates on the selectivity of oxidation. In the purely diffusion-controlled situation, there are two regions in the reaction solution—the near-surface region that borders the gas phase, and the liquid bulk that represents the majority of the solution volume. Here it should be reminded that lignins in alkaline media at 150–170 °C are generally quickly oxidized even without catalysts. Therefore, in the described reaction situation, the near-surface region is affected by quick oxidation to great extent—potentially to carbon dioxide and water—and this leads to the destruction of the desired aldehydes instead of their accumulation (Scheme 2). In this mode, the availability of oxygen in the solution bulk or on the catalyst surface is very limited. On the other hand, under the chemical control of the process, the concentrations of reactants and the aldehydes are similar across the entire liquid phase, and the aldehydes concentration can reach its practical maximum [69].

So, the selectivity of the heterogeneous processes of lignins oxidation in terms of the intended (but formally intermediate) products becomes higher as the kinetic mode gets closer to pure chemical control, and decreasing the wood-to-liquid ratio in the reactor intensifies mass transfer. This trend (the reduction of oxidation rate—potentially down to a complete halt—due to increasing lignin concentration) is observed in catalytic oxidation of soda cooking liquor from straw [70], lignosulfonates [43], and brown rotted pine wood [42]. The complete stoppage of the process is probably due to the lignin condensation type of side reactions that lead to the formation of an impenetrable polymeric film on the liquid-gas boundary that denies the access of oxygen for further reactions.

## 6. The Influence of Temperature on Yield of the Aromatic Aldehydes in Oxidation by Oxygen

The typical temperature range in which lignin oxidation by oxygen takes place is the same as with oxidation by nitrobenzene, 160–170 °C. Literature reveals no comprehensive insight into the influence of temperature on the effectiveness of oxidizing lignins into the aromatic aldehydes, and this Section summarizes the available fragmentary information.

First of all, the patent by Schoeffel [71] should be pointed out. It reports the possibility of high vanillin yields when oxidizing lignins at 200–220 °C: non-catalytic oxidation under these conditions produces twice as much aldehydes compared to the process at 160 °C. This result is supported by other literature data [68,72] (Table 3). Increasing the temperature of aspen wood oxidation from 160 to 190–200 °C leads to almost two times higher yield of vanillin and syringaldehyde (up to 31 wt. % based on lignin). This is close to the best results of catalytic aspen wood oxidation. An even greater increase of the aromatic aldehydes yield in aspen wood oxidation (10-fold, from 1.5 to 15%) is observed when raising the temperature from 110 to 160 °C [43,50].

Increasing the temperature of the catalytic oxidation of aspen enzymatic lignin from 130 to 170 °C also results in two times greater vanillin and syringaldehyde yield [39]. Similarly, our study of the temperature’s influence on the selectivity of pine enzymatic lignin oxidation showed that the vanillin yield increases monotonously in the temperature range 90–160 °C [73].

Increased yield of the aldehydes caused by higher temperatures can have many underlying explanations, and we will point out two of them. On the one hand, there is quite an obvious thermodynamical trend: at high temperatures, the equilibrium of depolymerization reactions favors their products; to some extent, this can explain the discussed temperature effect on aldehyde formation. On the other hand, there is a kinetic explanation that follows from the reaction mechanism discussed in Section 8: the side reaction of phenoxyl radicals dimerization can have near zero activation energy, but oxidation of the same radicals into the aldehydes has a significantly higher activation barrier [74,75]. Therefore, a higher temperature has a stronger accelerating effect on the reaction that leads to the intended products, and the side reaction of radical dimerization is thereby suppressed.

And speaking of lignin depolymerization at high temperatures, it is worth noting that this reaction is known to occur in alkaline delignification, i.e., when temperature and alkalinity are similar to those for oxidizing lignins into the aldehydes. Formation of free radicals with delocalization of uncoupled electrons was detected by electron spin resonance spectroscopy during alkaline delignification by Kleinert [76], probably phenoxyl radicals from homolytic cleavage of ether bonds between phenylpropane units. As will be discussed in Section 8, phenoxyl radicals are very important for generation of the aldehydes. In this context, one coincidence should be noted: in alkaline delignification, a certain portion of lignin (approx. 30%) is dissolved quickly compared to the rest of this polymer [41]. Approximately the same amount of lignin is typically converted to the aldehydes in oxidative processes [42]. This suggests that the high-temperature homolytic cleavage of lignin molecules may play the central role in the formation of the fragments suitable for oxidation into vanillin and syringaldehyde.

We should also mention two kinetic considerations that may have the opposite effect: increase of the aldehyde yield with lowering temperature. First, decreasing temperature biases the concurrence between the non-catalytic and the catalytic reaction pathways in favor of the latter because catalytic reactions generally have lower activation energy; and since the catalytic one is more selective, better aldehyde yields can be expected. Second, as explained in the previous Section, at lower temperatures the heterogeneous process is limited by strongly activated chemical reactions as opposed to easily activated diffusion, and chemical control is necessary for high yield of the aldehydes. While both these trends should increase the process selectivity in aldehydes at lower temperatures, in most works they do not manifest.

Still, the oxidation of Kraft lignin [77] in the temperature range 110–154 °C yields the highest vanillin amount (10.8 wt. % based on lignin) at 133 °C. The minimum temperature of 100 °C at which high yields of the aldehydes and their aceto-derivatives (17–19 wt. %) were still attained was reported for oxygen-alkali bleaching of technical-grade cellulose [78].

## 7. Kinetic Trends of Oxidation of Lignins

Understanding mechanisms and kinetics of chemical processes is crucial to the development of their technology. This Section discusses the kinetic trends of lignin oxidation, the next one focuses on the mechanisms behind these reactions, then the technological prospects of these processes will be discussed.

**Figure 2:**
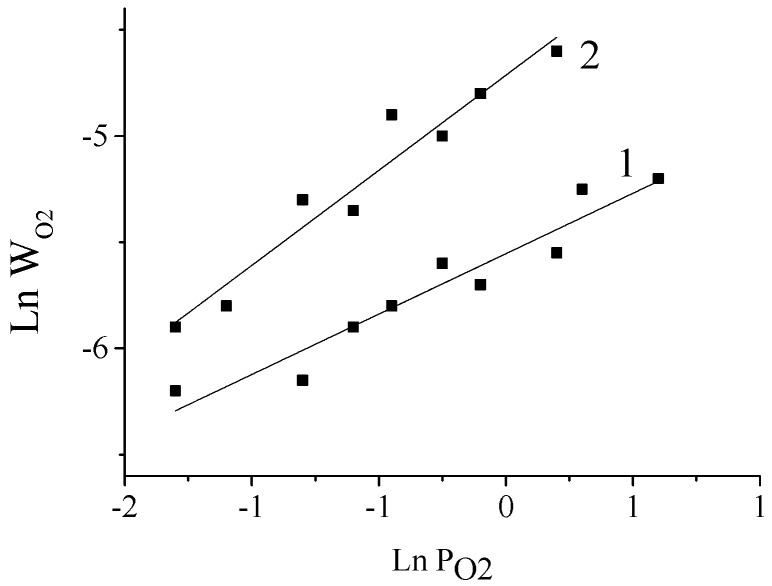
Logarithmic plots of oxygen consumption rate versus oxygen pressure in oxidation of aspen wood at 110 °C. (1) [OH^−^] = 0.003 M, reaction order in O_2_ 0.56 ± 0.04. (2) [OH^−^] = 2 M, reaction order in O_2_ 0.99 ± 0.10 (see references [44,67]).

There are works that studied the influence of oxygen pressure and hydroxide ion concentration on the rate of oxygen consumption in the oxidation of powdered aspen and fir wood under chemical control conditions in the temperature range 110–160 °C [44,67,68]. It was shown that at different reaction media alkalinity levels the process obeys the kinetics corresponding to different mechanisms of radical chain reactions. At low alkalinity (pH 7–9 [79]), chain initiation is caused via chain branching (hydroperoxide cleavage) and by oxidation of phenolate ions by oxygen (reaction order in OH^−^ is within the range 0–½). The chain branching mechanism also occurs in oxidation of non-extracted fir wood [68]. At higher alkalinity (pH 10–12.5), chain initiation proceeds via phenolate ion oxidation by oxygen and the process is governed by non-branching long-chain reaction kinetics (reaction order in O_2_ near ½, see Figure 2).

Further increase of NaOH concentration to 2 mol/L leads to the transition towards short-chain reaction kinetics with chain length as short as υ = 1–1.2, and the reaction order in oxygen increases to 1 [44,67]. This outcome is fully consistent with a general pattern of radical chain reactions [69,79]: chain length decreases as chain initiation rate rises (in this case, the rate of lignin’s phenolate anions oxidation).

The results of the reviewed publications [44,67,68,79] demonstrate that despite wood’s complex composition and irregular structure, basic trends of its oxidation can be analyzed and appear to concur with those in simple well-studies systems [80]. As for the connection between the discussed oxygen-related kinetic aspects of the process and the formation of vanillin, the following can be pointed out: wood oxidation enters the non-chain reaction mode due to the high rate of initiation via lignin’s phenolate ions oxidation at high alkali concentrations of 1–2 mol/L. Coincidentally, this strong alkalinity is necessary for selective oxidation of lignins into the aromatic aldehydes; the reasons for this coincidence will be analyzed in Section 8.

Other interesting patterns can be derived from studying the kinetics of lignosulfonate oxidation at the high temperatures typical for the technological conditions of vanillin synthesis. Analyzing the rate of vanillin accumulation with respect to the rate of oxygen consumption [43] reveals some trends concerning the influence of the process conditions and its selectivity towards vanillin.

**Figure 3:**
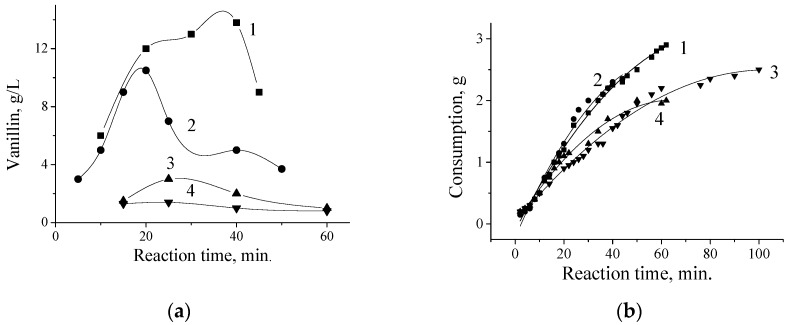
The influence of alkali concentration and pH on vanillin yield (**a**) and oxygen consumption (**b**) in lignosulfonate oxidation at 160 °C. Curve numbers correspond to the row numbers in Table 4 (1–5: 120 g/L NaOH; 2–6: 80 g/L NaOH; 3–7: 40 g/L NaOH + 300 g/L K_2_CO_3_, pH 10.5–10.8; 4–8: 300 g/L K_2_CO_3_, pH 10.3–10.0) (see reference [43]).

At 160 °C in the presence of catalyst, vanillin concentration in the reaction solution reaches the maximum (corresponding to 12 wt. % yield based on lignin) and then decreases upon further oxidation (Figure 3). This yield figure is close to the yield from nitrobenzene oxidation of lignosulfonates (16%, Table 1). Without a catalyst, the peak vanillin concentration is two times lower, but the oxygen consumption rate is practically independent of the catalyst amount (Figure 3, Table 4 rows 1, 4 and 5). Similar catalyst influence on the aromatic aldehydes yield was found with different lignin-containing feedstocks [42,43,51].

At 110 °C, catalysts (oxides of copper and silver) result in five-fold higher oxygen consumption rate and 1.4-fold higher vanillin yield (Table 4 rows 10–13). At 160 °C the oxygen consumption rate increase caused by the catalyst does not manifest, probably due to limitations by diffusion and the experimental accuracy: oxygen consumption (Figure 3) relative to vanillin yield in the highlighted experiments attains 13 mol/mol, while the reaction stoichiometry requires an order of magnitude smaller oxygen amount (see Section 8).

A temperature decrease from 160 to 110 °C leads to an almost two-fold decrease of the vanillin yield in the catalytic process (Table 4, rows 4 and 11), and the pertinent explanations were given in Section 6.

At 160 °C, a decrease of pH from 11 to 10 leads to almost complete suppression of vanillin formation, but does not have a significant effect on the rate of oxygen consumption (Figure 3, Table 4, curves and rows 7 and 8). The latter drops to zero only at veraciously lower pH 9–9.5 [43]. The difference between the minimum pH values at which oxidation of lignins per se takes place, and at which specifically vanillin formation occurs is the most important detail about these processes, something that was not known prior to that publication [43]. A similar result was observed in oxidation of Kraft lignin where vanillin stops forming when the reaction solution pH decreases from 14 to 10 [77]. This trend is very important for the formulation of the process mechanism as presented in the next Section because it demonstrates that vanillin formation requires higher alkalinity than it is necessary for the dissociation of lignin’s phenolic hydroxyls.

In this Section’s conclusion, we should again mention very high consumption of oxygen (and consequentially, of alkali) in the oxidation of lignosulfonates, around 13 mol per mol of vanillin. The situation is similar with other lignins: around 5% of the consumed oxygen goes into vanillin formation, and the rest of it is wasted to oxidation of some 20% of reaction solution’s organic compounds into carbon dioxide and carboxylic acids that bind alkali and decrease the system pH. The primary purpose of utilizing catalysts in oxidation of lignins is increasing the yield of the aromatic aldehydes to the level observed in oxidation by nitrobenzene, and in many cases it is quite successfully fulfilled (Table 1 and Table 2). However, there appears to be no research focused on catalysts that would considerably lower the consumption of oxygen and alkali.

## 8. The Mechanism of Oxidation of Lignins

There is a considerable array of literature devoted to oxidation of phenols; and for an introduction into a discussion on the mechanism of lignin oxidation, we will look into catalytic oxidation of *p*-cresol—the simplest alkyl-substituted phenol—into *p*-hydroxybenzaldehyde.

The reaction of hydroxyl radicals with cresol under radiolysis leads to phenoxyl radicals, and proceeds two orders of magnitude faster in alkaline media than in neutral [81]. Selective catalytic oxidation of *p*-cresol takes place only in alkaline media. To attain near 90% selectivity, superstoichiometric amounts of alkali are necessary (alkali: cresol = 2–3 mol/mol); in neutral media no oxidation occurs at all [82]. These trends of oxidation are quite general for any phenols, and have been known for a long time [73,74,83].

**Scheme 3:**
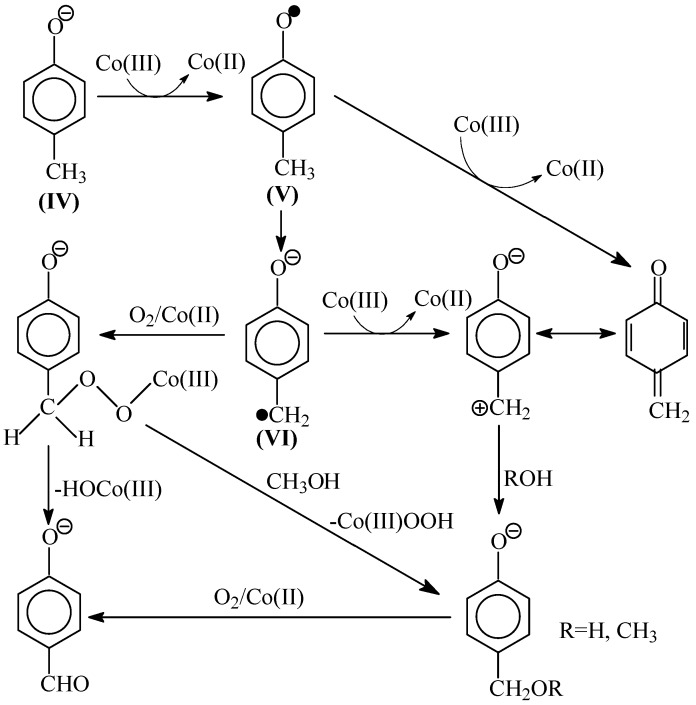
The mechanism of *p*-cresol oxidation according to Sheldon [83]. Curved arrows show the transformations happening to the oxidant.

Various published mechanisms can differ in details, but there are universally recognized principal stages of the process: formation of phenoxyl radicals, their transformation into quinone methides, and solvolysis of the latter into alcohols or aldehydes depending on the oxidation depth. In Scheme 3, Sheldon’s oxidation mechanism [83] is presented; it includes the listed intermediate species, and also—as should be pointed out in the interest of the later discussion—the CH-acid dissociation of a phenoxyl radical (**V**) into an anion-radical (**VI**).

The reactions of oxidation of alkyl phenols are obviously not completely selective. The principal side reaction pathway is the transformation of phenoxyl radicals: their dimerization (3) followed by oligomerization [74,84].

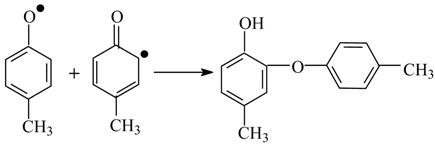
(3)

Phenoxyl radicals and quinone methides are widely discussed when describing the mechanisms of lignin oxidation in cellulose bleaching processes [85], as well as in the aromatic aldehydes synthesis [2,9,86,87,88]. Since the 1980’s, several hypotheses concerning the mechanism of lignins oxidation into the aromatic aldehydes were suggested. Before discussing their credibility, we will summarize the hereinabove discussed and well-known process trends that a theorized mechanism of lignin oxidation into the aromatic aldehydes in alkaline media needs to be able to explain or not contradict:

1. Oxidation of lignins into vanillin and syringaldehyde proceeds with oxidants of a different nature (nitrobenzene, copper oxide [30], oxygen with and without [71] catalysts) with high and similar selectivities (over 40 wt. % of aldehydes). It appears unlikely that the same outcome can transpire through different oxidant-dependent mechanisms in such a chemically complicated system. Therefore, a mechanism hypothesis should be universal with respect to oxidant nature.
2. Selective oxidation of lignins requires higher reaction medium alkalinity than is necessary for the dissociation of phenolic hydroxyls [43,77]. Thus, a vanillin formation mechanism may include acidic dissociation of an intermediate that is less acidic than lignin’s phenolic hydroxyls.
3. Production of vanillin and syringaldehyde from lignins is accompanied by the formation of the corresponding aceto-derivatives as side products.
4. Vanillin, syringaldehyde, and their aceto-derivatives are produced in smaller amounts in lignin alkaline hydrolysis without oxidants. This indicates that lignin oxidation and lignin alkaline hydrolysis may have common stages, and these should probably be the final stages.

The first known hypothesis about the mechanism of vanillin formation does not relate to oxidation reactions. It is known that alkaline hydrolysis of lignosulfonates produces vanillin (up to 5.9 wt. % based on lignin) without oxidants [1,24]. In 1936, Hibbert [26] suggested that this vanillin formation is caused by alkali-catalyzed retroaldol reaction of the α-hydroxy-γ-carbonyl structure of a phenylpropane lignin structural unit:

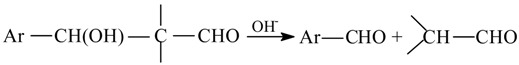
(4)
where Ar is 3-methoxy-4-phenoxy anion. This mechanism is good at describing oxidantless alkaline hydrolysis of lignin. First, it can explain the formation of acetovanillone and acetosyringone as the byproducts of lignin alkaline hydrolysis [24,26] by similar cleavage of an α-carbonyl structure:
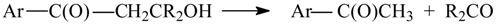
(5)

Second, Reactions (4) and (5) require strong alkalinity because formation of the enolate anion necessary for aldol condensation and cleavage reactions [89] is caused by further dissociation of a phenolate anion of a lignin PPU: 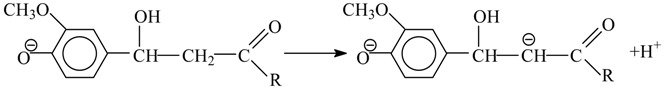
(6)
p*K* values of model lignin compounds attain 9–10 and more [90,91]. Obviously, the dissociation constant of Reaction (6) is several orders of magnitude less than the figure for the PPU phenolic hydroxyl. Therefore, effective conduction of the Reactions (4) and (5) is only possible in media with considerably higher alkalinity than what is necessary for acidic dissociation of lignin phenolic hydroxyls (7), and for their fast oxidation when oxidants are present.
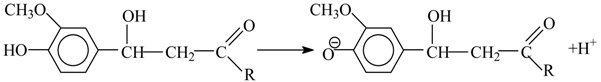
(7)

Vanillin yield without oxidants is modest which agrees with relatively low content of carbonyl groups in lignins [92]. So, Hibbert’s suggested pathway of the lignin alkaline hydrolysis is in good agreement with experimental facts about oxidantless vanillin formation, and with general patterns of retroaldol reaction. Hibbert’s hypothesis agrees with three out of four hereinabove defined requirements for a mechanism of lignin oxidation into the aromatic aldehydes. It is naturally tempting to extrapolate this productive hypothesis towards oxidative lignin cleavage. This would mean limiting the role of an oxidant to formation of the carbonyl group necessary for the retroaldol reaction which finalizes the aldehyde production process. However, for 60 years there were no attempts at using the Hibbert’s hypothesis as the basis for elucidating the mechanism of oxidative lignin cleavage.

Now we will briefly consider hypotheses (**H1**–**H3**) about the mechanism of lignin oxidation into the aromatic aldehydes that were formulated in 1980’s and are frequently cited in many reviews to the present day.

**Hypothesis** **1** **(H1).** Schultz’s hypothesis [93,94] on the mechanism of lignin oxidation by nitrobenzene via abstraction of an electron and then of a proton from the benzylic hydroxyl of a lignin structural unit, instead of an attack on the phenolic hydroxyl. β-cleavage decay of the remaining benzoxyl radical leads to vanillin:

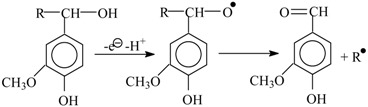
(8)

Schultz’s hypothesis does not agree with the mechanism requirements **3** and **4**.

**Hypothesis** **2** **(H2).** A hypothesis about the mechanism of lignin oxidation by oxygen via formation of 1,2-dioxetane ring and its subsequent cleavage leading to vanillin and a carboxylic acid [9]:

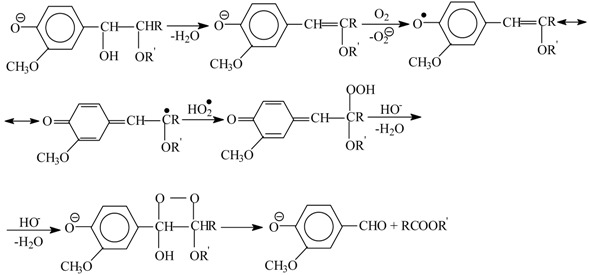
(9)

This mechanism is adopted from discussions on chemistry of cellulose oxygen bleaching [95,96,97] in order to explain vanillin formation [9], and is cited in many reviews. Its principal drawback is that it cannot be extrapolated towards two more selective oxidants: nitrobenzene or copper oxide.

**Hypothesis** **3** **(H3)** **A hypothesis about the mechanism of lignin oxidation that involves a retroaldol reaction step.** Based on various experimental results [86,87,88,98] and discussions in literature [85,95,99,100,101], a mechanism hypothesis for formation of vanillin in lignin oxidation was developed in 1997–2004. It unites the universally recognized lignin chemistry principles about phenoxyl radicals and quinone methide intermediates with the concept of retroaldol reaction as the final process step. To keep the presentation of this hypothesis [86,87,88,98] brief, it will be rendered as oxidation of eugenol as an example model compound.

The oxidation begins with abstraction of an electron from a phenolate anion:
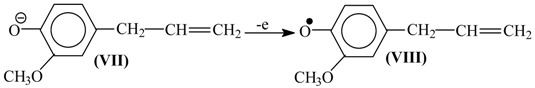
(10)

Disproportionation of these primary radicals (**VIII**) leads to a quinone methide (**IX**):
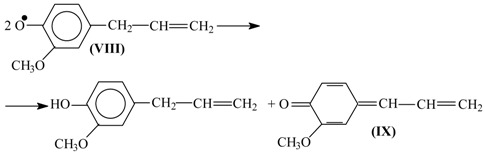
(11)
and nucleophilic addition of a hydroxide ion to the latter creates the coniferyl alcohol structure (**X**).
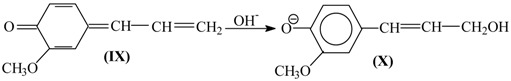
(12)

It should be noted that formation of the quinone methide (**IX**) out of the phenoxyl radical (**VIII**) is also possible via acidic dissociation of the latter with the following single-electron oxidation of the resulting radical-anion. This possibility was discussed by Sheldon (Scheme 3 [83]) and in our works [86,87,102].

Subsequent oxidation of coniferyl alcohol (**X**) is analogous to Reactions (10)–(12) and results in the γ-carbonyl group (**XI**). Retroaldol reaction of the α-unsaturated aldehyde (**XI**) yields vanillin (**XIII**):
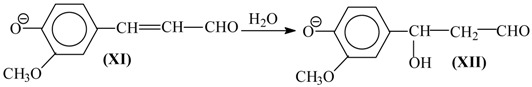
(13)

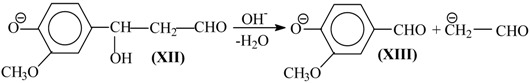
(14)

Step (14) proceeds through C-H acidic dissociation of the phenoxyl anion (**XII**) in a fashion similar to Equation (6). This requires strong alkalinity, and if the medium alkalinity is not sufficient for this, then retroaldol reaction will fail, and instead oxidation of (**XII**) into byproducts (perhaps, into ferulic acid) will take place. The latter takes place, for example, in oxidation of lignosulfonates at pH 10.0–10.8 (Figure 3). The assumed ferulic acid formation produces new carboxylic acidic protons that neutralize the alkali and decrease the pH. Speaking of alkali consumption, other unintended oxidation reactions (e.g., caused by the excessively reactive intermediate products of molecular oxygen reduction, more about them later) will also eventually generate acidic byproducts (other carboxylic acids and CO_2_) that bind alkali.

The suggested mechanism allows explaining the formation of the aceto-derivatives of the intended aldehydes by assuming the addition of a hydroxide ion into the α-position of the quinone methide (**IX**) with its subsequent oxidation and eventual retroaldol reaction of the resulting α-oxo-β-unsaturated structure (**XVII**) (15):
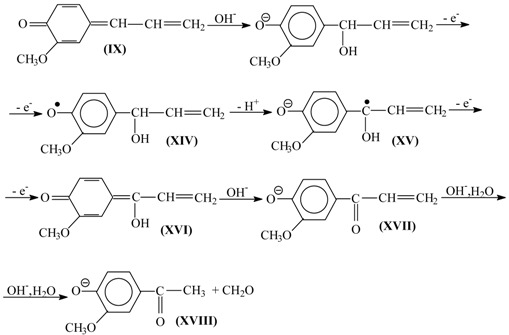
(15)

Reaction (15) demonstrates formation of the quinone methide (**XVI**) by acidic dissociation of the phenoxyl radical (**XIV**) with the subsequent oxidation of the anion-radical (**XV**), instead of the disproportionation (11) of (**XIV**).

Hydroxide anion addition into the α-position of a phenylpropane unit is less thermodynamically favorable than addition into the γ position since the former disrupts the π conjugated chain [85,102]. Thus, under thermodynamic reaction control, aceto-derivatives will be a minor byproduct. According to literature data [85], nucleophilic addition to quinone methides is controlled by thermodynamics. However, one exception is known to the pattern concerning the high ratio of the aldehydes to their aceto-derivatives in the process products: In oxidation of birch phloem (inner bark), peak yields of syringaldehyde and acetosyringone are almost equal [103].

We should point out one important aspect of the mechanism (10)–(15) as well as of many other radical reactions involved in lignin transformations: Interconversion between a phenolate anion and a phenoxyl radical can be fast and reversible. This kind of electron transfer between semiquinone radicals and hydroquinone anions proceeds with rates close to that of diffusion [74,104]. This means that the ratio between the molecular species along the oxidation chain—eugenol, coniferyl alcohol, coniferyl aldehyde, vanillin—can be controlled to a significant degree by thermodynamics of the electron exchange in various combinations of their phenolate and phenoxyl radical forms. These reactions may determine high selectivity of vanillin formation (more specifically, low rate of vanillin oxidation).

For example, in a Steelink’s study, in oxidation of syringyl alcohol, oxidation of syringaldehyde is only observed when the alcohol conversion of over 90% is reached [105]. Redox potentials of single-electron oxidation of eugenol, isoeugenol, coniferyl alcohol, and other phenols were derived in [106]; it shows that donor substituents decrease the potential, whereas electron-withdrawing substituents increase it. This means that in oxidation under thermodynamical control, vanillin is a more stable species than eugenol and coniferyl alcohol. Vanillin cannot be oxidized by the phenoxyl radical of eugenol. On the other hand, an otherwise (16) generated phenoxyl radical of vanillin can revert (17) to vanilloxide anion by oxidizing a phenolate ion of the predecessor compounds (lignin, eugenol, coniferyl alcohol, etc.) into a corresponding phenoxyl radical of the latter:
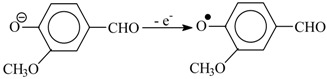
(16)

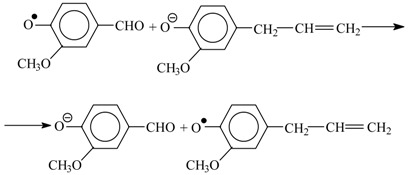
(17)

On one hand, the presence of the electron-withdrawing carbonyl group in the *para*-position relative to the phenolic hydroxyl in the aromatic aldehydes contributes to their high yields in oxidation of lignins (by increasing the redox potential of the former). On the other hand, the same withdrawing substituent causes a negative effect related to increased phenolic acidity: the p*K*_a_ of vanillin is 7.2 [107] which is lower than the phenolic p*K*_a_ of lignins by approximately 3 [91]. So, in weakly alkaline media (pH 8–9) vanillin is dissociated, whereas lignin is not. In this situation, vanillin—the intended product—will be oxidized faster than lignin and therefore will be unstable. Thus, an alkali excess is necessary for the product stabilization that is achieved via lignin phenolic dissociation. The similar difference between the acidities of *p*-hydroxybenzaldehyde and *p*-cresol [90] explains why superstoichiometric amounts of alkali are necessary for selective oxidation of the latter [83].

The conducted analysis shows that the mechanism we presented for vanillin and syringaldehyde formation in lignin oxidation in alkaline aqueous media has certain advantages over other known hypotheses. It allows explaining all of the hereinabove outlined patterns of the process, and also a number of experimental results (**R1**–**R4**) obtained in testing this mechanism’s validity [86,88,98]:

**Result** **1** **(R1).** Influence of pH on the kinetics of vanillin formation shows the same trends in the oxidation of vanillideneacetone, eugenol, and lignin; this supports the role of retroaldol reaction in the test processes.

**Result** **2** **(R2).** Coniferyl alcohol was detected as an intermediate in the process of eugenol oxidation.

**Result** **3** **(R3).** The compositions of the aromatic products from oxidation of guaiacylpropanol and guaiacylethanol were fundamentally different; this fact is consistent with the proposed mechanism and cannot be explained in terms of other well-known hypotheses.

**Result** **4** **(R4).** The old industrial process of eugenol to vanillin conversion included a separate and very slow step of eugenol to isoeugenol isomerization followed by isoeugenol oxidation [8]. We showed that selective catalytic oxidation of eugenol to vanillin can be carried out directly without the isomerization step. This fact supports the suggested mechanism, because the steps (11) and (12) lead to the double bond migration from α- to β-position during the oxidation instead of the separate eugenol isomerization step.

Based on the mechanism of the aromatic aldehydes formation (10)–(15), several conclusions (**C1**–**C4**) and recommendations about the choice of catalysts and the process conditions for lignin oxidation by oxygen can be made.

**Conclusion** **1** **(C1).** Phenolate anions are among the most easily oxidized functional groups in organic chemistry, and oxidation of lignins in alkaline media proceeds with quite high rates even without catalysts. For this reason, the principal role of catalysts in this process is increasing its selectivity instead of the oxidation rate. To this end, assuming stepwise interaction of the catalyst with phenolate anion and then with oxygen, a catalyst’s redox potential needs to be as low as possible while being able to oxidize the phenolate anion. The optimal redox potential for a catalyst of lignin oxidation can be qualitatively characterized: it needs to be between the redox potentials of single-electron oxidation of vanillin and of, e.g., eugenol. In the context of the mechanism (10)–(15), the oxidized form of an optimal catalyst withdraws an electron from phenolate anions that are predecessors to vanillin (10)–(12), and should have sufficiently low oxidation potential to not affect more deeply oxidized ligneous phenolic species and vanillin. The reduced form of the catalyst should not lead to formation of free active oxygen-containing radicals upon reaction with molecular oxygen.

A traditional and quite selective catalyst (also, a stoichiometric oxidant [30]) of the process is copper oxide, it has modest electrode potential (−0.16 V for CuO/Cu_2_O redox pair at pH 14) [108]. Sliver oxide has higher potential (+0.34 V at pH 14) [108], and using it in lignin oxidation results in higher vanillic acid yield compared to copper oxide [24], i.e., oxidation of vanillin takes place. This comparison shows that an electrode potential +0.34 V in alkaline media is already excessive for a selective catalyst. As far as vanillin stability is concerned, it is worth mentioning that molecular oxygen in alkaline media has a redox potential of +0.40 V, the figure is about the same for superoxide O_2_^−^, while hydroxyl radical HO**^●^** has an electrode potential +2.0 V in alkaline media [108]. All these figures are too high in terms of vanillin preservation. Therefore, a good catalyst of the process might fulfill the important role: decreasing the impact of oxygen and its radical forms on the process selectivity by consuming them via reactions with reduced catalyst species. However, an important conclusion to the discussion about the suitable catalyst electrode potential should be that the catalytic reaction cycle with any catalyst might occur without formation of unoccupied reduced catalyst sites as intermediates. This type of mechanism would reduce the influence of the catalyst redox potential on its ability to maintain the process selectivity (even mild oxidants may prove to be good catalysts of vanillin oxidation if the mechanism does not actually involve redox cycling of unoccupied catalytic sites).

**Conclusion** **2** **(C2).** Another way of improving the selectivity is suppression of the phenoxyl radicals dimerization pathway (3) in favor of their oxidation (11) and (15). The most straightforward approach here is to increase temperature (see Section 6) [73,104,109].

**Conclusion** **3** **(C3).** It is worth noting that the described mechanism of selective lignin oxidation into vanillin involves radicals but is not essentially a chain reaction: the phenoxyl radical (**VIII**) formed at the initiation stage (10) is transformed into a molecular product without chain propagation. Therefore, the mechanism (10)–(14) and (15) can be considered chain termination. Non-catalytic oxidation by oxygen typically proceeds as chain reactions involving alkoxyl, hydroxyl, and peroxyl radicals that are too active to maintain the process selectivity. Therefore, the optimal catalyst and process conditions need to suppress the oxidation chain propagation. It was noted in Section 7 that apparent chain length in the process of powdered aspen wood oxidation decreases to as low as unit at alkali concentration 1–2 mol/L. At this point, we can name three phenomena via which high reaction media alkalinity contributes to better accumulation of the aldehydes: (1) promotion of retroaldol reaction, (2) extension of phenolic dissociation to lignin molecules as opposed to only the intended products, (3) suppression of oxidation chain propagation by increasing the chain initiation rate.

**Conclusion** **4** **(C4).** Based on the offered mechanism, it is not difficult to estimate the minimum oxygen amount necessary to obtain one mol of vanillin. e.g., in oxidation of coniferyl alcohol, it is ½ mol O_2_ per mol of vanillin. In oxidation of lignin, this oxygen requirement may be even lower since lignin is formed from coniferyl and sinapyl alcohols via condensation after their single-electron oxidation into the corresponding phenoxyl radicals [22].

As was previously noted, in practice, oxygen consumption is immensely higher, alkali consumption is therefore much higher than theoretically needed too. Decreasing the consumption of these substances is an unresolved and clearly attention-worthy problem. Detailed insight into the nature of this problem lies outside the scope of this review, but at a glance, a few factors contributing to it can be mentioned.

The described mechanism depends on a series of single-electron oxidation attacks on phenolate anions, and when an oxygen molecule performs such attack, a superoxide anion-radical O_2_^−^ is generated. The latter, being an anion, will experience electrostatic hindrance when trying to attack the next phenolate anion, and is more likely to be instead wasted on some particle irrelevant to the formation of the intended product. i.e., while an oxygen molecule could perform up to four acts of single-electron oxidation before being fully reduced, not all of this capacity is guaranteed to go towards the aldehydes formation, hence low selectivity based on oxygen. Overcoming this problem would require a challenging job of finding a catalyst which ensures not only binding of all intermediate oxygen species (as described above), but also their selective participation in lignin oxidation. Another requirement for high selectivity is confining the action of oxygen to the surface of this catalyst (decreasing the contribution of the unselective non-catalytic process). Finally, certain components of wood (e.g., extractives, hemicelluloses) can be removed prior to the process so that additional oxygen is not spent on their oxidation. However, the latter two factors relate more to the process implementation design, and as such will be discussed more in the following Section.

## 9. Development Prospects for the Technology of the Aromatic Aldehydes Production from Lignins

The concluding Section reviews the data on the results concerning technological aspects of lignin oxidation.

### 9.1. Oxidation of a Lignosulfonate Solution or Aspen Wood Slurry in a Flow Reactor System

**Scheme 4:**
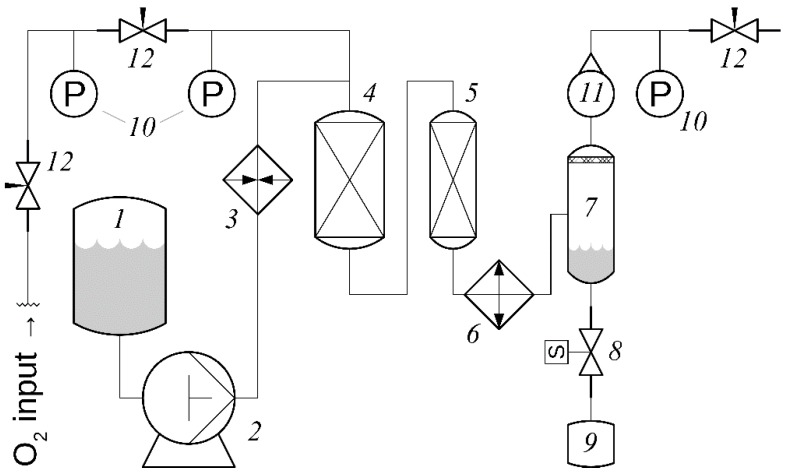
The flow system for lignosulfonate oxidation. (1) initial reaction solution tank; (2) plunger pump (1–10 L/h, 100 kgf/cm^2^); (3) heater of liquid (0.25 L); (4) reactor (1.0 L); (5) reactor (0.25 L); (6) cooler; (7) separator; (8) solenoid valve; (9) reaction mass sampling vessel; (10) manometers; (11) rotameter; (12) regulating valves.

The kinetic trends of lignosulfonate oxidation in a shaking batch reactor [43] described in the previous Sections were used to devise a similar process of continuous operation [50,110]. The oxidation of lignosulfonates was carried out at 170 °C with operating pressure around 1 MPa using an installation with two fixed-bed catalytic reactors connected in series completely filled with cupric oxide catalyst (Scheme 4). The reaction solution and oxygen were fed into the reactors from top to bottom, achieving falling film flow of reactants along the surface of the catalyst grains.

**Figure 4:**
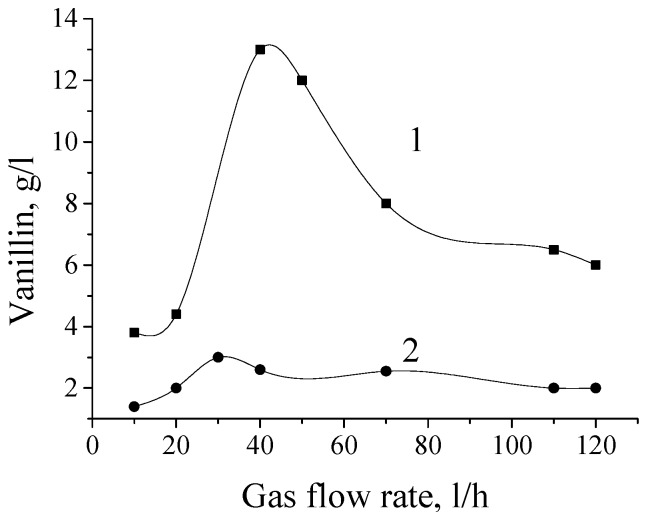
The influence of oxygen flow rate on yield of vanillin (1) and syringaldehyde (2) in oxidation of sulfite liquor in the flow reactor system. 170 °C, 0.9 MPa, 210 g/L of sulfite liquor dry mass, 150 g/L of NaOH, liquid flow rate 1.8 L/h (see reference [110]).

The rate of oxygen flow has a significant effect on vanillin yield in oxidation of sulfite liquor: increasing it by the factor of 2–4 leads to three-fold improvement of the vanillin concentration (Figure 4). This effect can be explained by assuming that the higher gas amount displaces the liquid, thus leading to a thinner liquid layer on the outer surface of the catalyst grains. This in turn results in a shorter diffusion path through the liquid that oxygen has to travel to reach the catalyst surface and/or deep reactant-rich liquid region. Overall, this leads to higher oxidation selectivity, as was described in detail in Section 5. At flow rates above 40 L/h, the yield decrease can be explained by lower liquid residence time inside the reactors, which is insufficient for attaining adequate oxidation extent. Maximum aldehydes yield attains 9.9 wt. % based on the lignin, and the liquid reaction mass hourly space velocity through the reactors attained 1–2 h^−1^. For comparison, we obtained 12 wt. % yield of the aldehydes in a batch reactor using oxygen, and oxidation of lignosulfonates by nitrobenzene yields up to 16 wt. % of the aldehydes based on the lignin.

It is well known that in catalytic processes that involve gaseous, liquid, and solid phases only the catalyst grain outer surface is primarily active [111,112]. Therefore, the high catalyst surface area necessary for a good yield of the products can be practically achieved only by decreasing the catalyst grain size. This would lead to high hydrodynamic flow resistance which constrains the maximum possible flow system efficiency.

When discussing practical implementations of catalytic lignin oxidation, we should point out another factor that restricts utilization of a catalyst’s inner surface in these processes. The aldehydes resulting from oxidation of lignins can be oxidized in lignin-free media (it should be emphasized, in lignin-free media) with rates that are comparable to the rate of their formation [43,54,113]. This means that under flow conditions there needs to be a very tight distribution of catalyst contact time for the reacting molecules. It is quite obvious that the contact time for reactant particles that are carried along the catalyst outer surface is considerably shorter than for those that diffuse inside the catalyst grains. Therefore, the aldehyde molecules that form on the catalyst inner surface are likely to transform into deep oxidation products before diffusing out into the liquid flow.

Oxidation of finely powdered (less 0.1 mm) aspen wood slurry in the same flow installation (Scheme 4) was accompanied by complete wood and cellulose dissolution. Yields of the aldehydes up to 37% based on lignin were attained [50]. This corresponds to 80–85% of the selectivity in oxidation by nitrobenzene (Table 1). This aldehyde yield is several times greater than anything attainable with technical lignins (Table 1 and Table 2). However, utilization of native lignin oxidation for production of the aldehydes alone is likely not an economically effective endeavor because potentially valuable cellulose and hemicelluloses are lost in the oxidation process. Moreover, oxidation of these carbohydrates requires additional consumption of oxygen (and consequentially, of alkali). Thus, an economically viable approach to produce vanillin and syringaldehyde must be based around comprehensive wood processing that involves production of value-added chemicals from cellulose and hemicelluloses.

### 9.2. The Prospects of Comprehensive Wood Processing into the Aromatic Aldehydes and Valuable Carbohydrate-Derived Products

It was mentioned earlier that vanillin production by oxidation of lignosulfonates dominated the industry in 1950–1970’s. With the exception of “Borregaard LignoTech”, this technology was universally supplanted by the guaiacol-glyoxylic synthesis. There are many reasons for this shift, and firstly an environmental one has to be stated: production of one tonne of vanillin from lignins results in over 100 m^3^ of waste water [8]. On the other hand, waste water volumes largely depend on the technology development level: for example, in the pulping industry this amount used to attain 60–160 m^3^ per tonne of cellulose [114], but in modern implementations it is near zero. Another considerable drawback of lignin oxidation is high alkali consumption (that attained 7–14 kg per kg of vanillin [43,44,115]).

Both these problems stem from low vanillin yield from lignosulfonates, 7–12 wt. %. Considerably higher amounts can be obtained from brown rotted pine wood, 20% (Table 1 and Table 2). This 1.5–3 fold gain of the aldehyde yield from enzymatic lignin as opposed to lignosulfonates is a clear advantage of the former. However, the vast natural resources of brown rotted wood are sparsely dispersed in nature, and thus are not a viable industrial feedstock.

Enzymatic technologies of biobutanol production are in intense development, and their byproduct is a substance that is quite similar in structure and properties to lignin of brown rotted wood [116,117,118]. It was shown that the enzymatic lignin from biobutanol production can be a promising feedstock for production of vanillin and syringaldehyde [39].

Even higher vanillin yields can be obtained in oxidation of softwood native lignins, 23–28 wt. % based on lignin [29,42]. However, for the reasons stated herein above, production of vanillin and syringaldehyde from native lignins can only be economically efficient if the carbohydrates of wood are also processed into valuable compounds.

**Figure 5:**
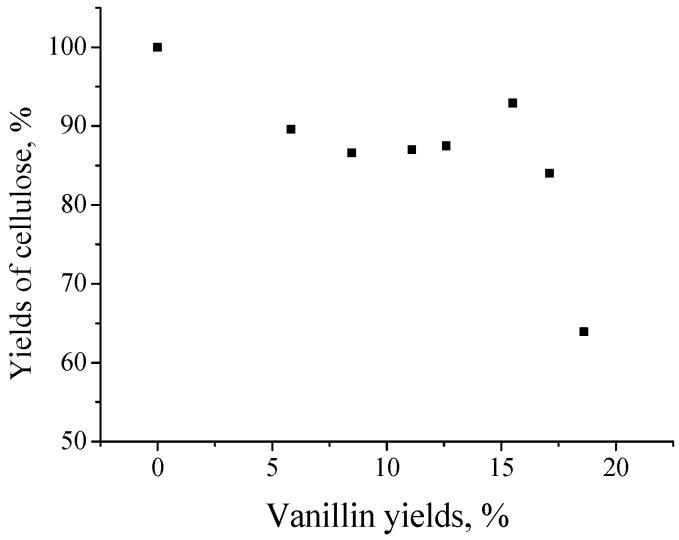
Vanillin and cellulose yields in pine wood catalytic oxidation in alkaline aqueous media at 160 °C [121].

Apparently, the possibility of obtaining the aromatic aldehydes and cellulose together by oxidation of hardwood and softwood was first described in patents [119,120]. Catalytic oxidation of pine wood into vanillin and cellulose with subsequent enzymatic hydrolysis of the latter into glucose was recently explored in more detail [121]. The maximum attained vanillin yield is 18.6 wt. % based on the initial lignin (Figure 5). Cellulose yield varies between 64% and 93% (based on its initial content in the wood), depending on oxidation conditions. The best compromise achieved is 17 wt. % vanillin (based on the initial lignin) and 84% cellulose (based on the initial cellulose in wood).

Thus, there exist certain conditions under which the catalytic oxidative production of vanillin also fulfills the role of oxidative wood delignification, and it is quite selective in this regard. The cellulose obtained in this manner may not be suitable for high-quality paper production, but there are no restrictions for its further processing into glucose, levulinic acid, 5-hydroxymethylfurfural, and other products. These results [119,120,121] reveal the possibility of implementing a novel approach to catalytic wood conversion into an assortment of fine chemicals.

**Scheme 5:**
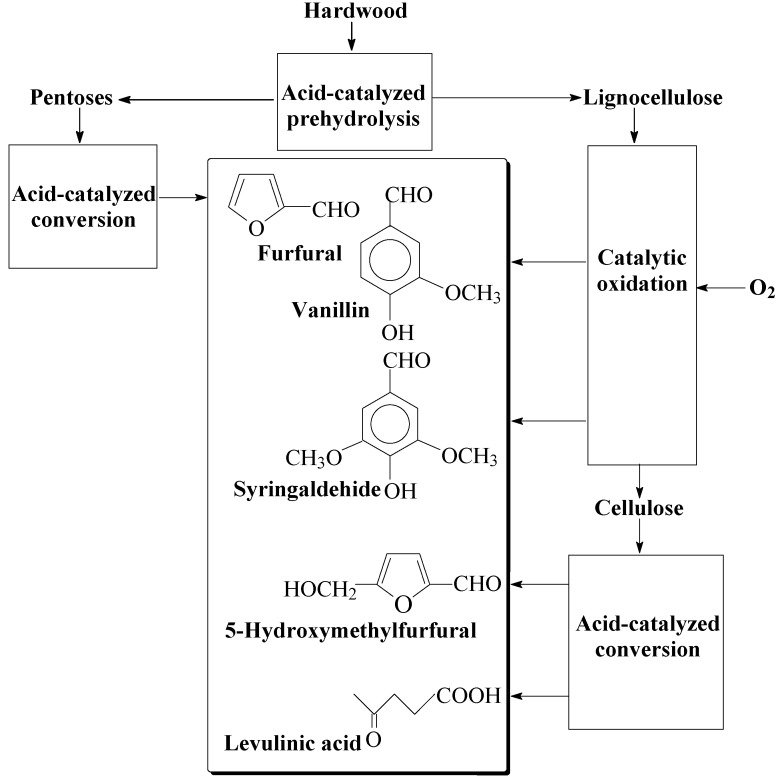
Comprehensive processing of hardwood involving oxidation of lignin.

Effective comprehensive wood processing into these fine chemicals should in general include three principal stages: (1) prehydrolysis and hemicellulose processing; (2) oxidative conversion of the lignocellulosic residue into vanillin, syringaldehyde, and cellulose; (3) processing the cellulose into glucose, levulinic acid, 5-hydroxymethylfurfural, and other products (Scheme 5). Depending on the type of wood, these stages may be preceded by removing wood extractives, phenols, etc.

The stage of hemicellulose removal allows both reducing consumption of the reactants during catalytic oxidation, and obtaining additional quantities of furfural or levulinic acid [122]. We are currently developing other ways of decreasing the oxygen and alkali consumption that will soon be revealed. It should be noted that softwood and hardwood considerably differ not only in structure of lignin but also of hemicelluloses: softwoods contain 5–9% of pentosans as opposed to 16–24% in hardwoods (aspen and birch respectively) [123]. So, softwoods are not particularly suitable for furfural production, and birch is preferable to aspen.

Syringaldehyde that forms together with vanillin in oxidation of hardwood lignins should preferentially be obtained from birch wood as well, as it produces better syringaldehyde to vanillin ratio than aspen [50,51]. Technical lignins are not very suitable for syringaldehyde production with the exception of lignins from enzymatic hydrolysis [39]. The problem of separating the product mixture of syringaldehyde and vanillin can be solved, for example, via precipitation of a syringaldehyde aldimine [124,125,126,127,128].

All the mentioned products with the exception of furfural are sparingly volatile and are typically isolated from aqueous solutions by extraction, an overview of the relevant methods can be found in [126,128].

Decreasing the volumes of the waste water from the process may be accomplished together with alkali regeneration: Similarly to procedures used in Kraft pulping [129], organic contaminants are oxidized in the liquid phase or combusted and the remaining soda ash is caustified. Residue from alkaline oxidation of native lignins can be used as pigments for timber and chipboards, as plant growth stimulators in farming, etc. [130]. Unlike similar substances derived from coal or lignosulfonates, these products do not contain carcinogens or sulfur compounds.

As a conclusion to this Section, Table 5 presents the data on global production and prices of the relevant products. The global vanillin market amasses 15–20 thousand tonnes annually at the price 10–15 USD/kg, and the world’s production capacity outweighs the market demand [131]. Global price of furfural is around 1–2 USD/kg, levulinic acid is worth around 3 USD/kg. Levulinic acid is primarily used in the polymer industry for synthesis of diphenolic acid and porophors [132,133]. Syringaldehyde is potentially the most valuable product among the discussed ones, serving as a precursor to 3,4,5-trimethoxybenzaldehyde the price of which exceeds 25 USD/kg. 3,4,5-trimethoxybenzaldehyde is probably ordinarily synthesized from vanillin [16] and is used in production of pharmaceuticals and other speciality chemicals.

Market value estimates of the products from one tonne of birch wood processed according to the suggested approach attains 1800–2400 USD and potentially even more if production of xylitol is implemented. For comparison, the value of the cellulose alone obtained from that wood is 250–300 USD.

Significant portion of the gross product value depends on syringaldehyde and xylitol, which are exclusive to hardwood processing. Assuming sufficient demand for these products, the processing of birch wood will be more economically efficient than the similar processing of softwoods.

## 10. Conclusions

The review of literature data reveals that properly organized processes of catalytic oxidation of various lignins are only insignificantly (10–15%) inferior to oxidation by nitrobenzene in terms of yield and selectivity in vanillin and syringaldehyde. Traditionally applied copper, manganese, and cobalt catalysts insignificantly differ in the selectivity of oxidation, and they do not perform appreciably worse than later developed catalytic systems.

Heterogeneous catalysts based on copper oxide were used (and are currently used [115]) in industrial lignosulfonate oxidation, and will probably remain the most suitable for the processes of this kind. These catalysts have numerous advantages in lignin oxidation processes: low cost, resistance to poisoning, high selectivity, ease of separation from the aromatic aldehydes (by extraction) and cellulose (based on density difference).

Among the as yet unresolved problems in oxidation of lignins into the aromatic aldehydes, very high consumption of oxygen (and consequentially, of alkali) is highlighted—over 10 mol per mol of obtained vanillin. While the presently known catalysts allow achieving yields of the aldehydes that are very close to the results of oxidation by nitrobenzene, there is still an unexplored challenge of finding catalysts that will also markedly decrease the consumption of oxygen and alkali.

Among the discussed hypotheses of the aromatic aldehydes’ formation mechanism, the most convincing one is the pathway that involves formation of the aldehyde molecule by retroaldol reaction of substituted coniferyl aldehyde (or β-oxycarbonyl structure). The latter structure is generated from lignin by sequential acts of single-electron oxidation of phenolate ions. This mechanism does not depend on the oxidant nature, and hence is a more general pathway compared to other hypotheses [9,93]. This mechanism explains the two most important trends of lignin catalytic oxidation by oxygen: formation of the aceto-derivatives of the intended aldehydes as byproducts, as well as the great difference between minimal pH needed for oxygen consumption as such (>9), and formation of vanillin as a product (>11).

The possibility of producing both cellulose and the aromatic aldehydes in one-stage catalytic oxidation of wood [119,120,121,122] is discussed. These results pave the road towards a novel technology of comprehensive wood processing into vanillin, syringaldehyde, and carbohydrate-derived products like levulinic acid and furfural. Native lignins yield 2–3 times more of the aromatic aldehydes compared to lignosulfonates, and it is a strong argument supporting the technology development of the direct wood conversion into platform chemicals. The carbohydrate components of wood can be processed by various known methods, when hemicelluloses are removed prior to the catalytic oxidation of wood, and when the cellulose is isolated afterwards.

Syringaldehyde is a possible precursor to 3,4,5-trimethoxybenzaldehyde and pharmaceuticals derived from the latter, and it is not produced in industry by lignin oxidation. Synthetic approaches to syringaldehyde production are more costly compared to vanillin [16], while production of the former by hardwood lignin oxidation can be cheaper compared to lignin-derived vanillin. This is because of a higher yield of syringaldehyde from hardwood lignins (up to 30 wt. % of lignin) than of vanillin from softwood lignins (up to 20%).

Three major industrial technologies of vanillin synthesis were implemented throughout history: oxidation of eugenol (the first half of the twentieth century), oxidation of lignosulfonates (1940–1970’s) [8], and the contemporary technology with guaiacol and glyoxylic acid. The glyoxylic technology currently dominates the market with the exception of Borregaard that utilizes both lignin oxidation and the glyoxylic method to manufacture vanillin, and this suggests that both technologies are comparable in economic viability.

The discussed results and concepts can serve as the basis for developing a technology for production of the aromatic aldehydes from renewable plant matter that may supersede the current technology of vanillin production from petrochemicals.

## Figures and Tables

**Table 1:** Nitrobenzene oxidation of various lignins into the aromatic aldehydes.

Lignin Type	Yield, wt. % of the Lignin		Reference No.
	Vanillin	Syringaldehyde	
Silver fir wood (*Abies alba*)	24.3	-	[29]
Norway spruce wood (*Picea abies*)	27.5	0.06	[23]
White spruce wood (*Picea glauca Voss*)	20.1	-	[22,30]
Quaking aspen wood (*Populus tremuloides Michx*)	12.	30.	[30]
Quaking aspen wood (*Populus tremuloides Michx*)	11 *	53 *	[31]
Birch wood (*Betula pendula Roth*.)	12	35	[29]
Norway maple wood (*Acer platanoides*)	13	37	[32]
Milled wood lignin of Loblolly pine (*Pinus taeda* L.)	26.6	-	[33]
Organosolv ethanol lignin **	6.7	17.0	[32]
Kraft softwood lignin	13.1	0.6	[32]
Kraft hardwood lignin	5.3	7.9	[32]
Sulfite softwood lignin	16.5	trace	[32]
Sulfite hardwood lignin	6.1	10.1	[32]
Brown rotted spruce wood	13.9	-	[34]
Pine soda lignin	5.2	-	[10]
Japanese cedar soda-anthraquinone lignin (*Cryptomeria japonica*)	8–11.2	-	[35]
Indulin AT commercial pine kraft lignin	10.9	-	[35]
Pine prehydrolysis liquor precipitate	8.00 ***	-	[36]
Klason hydrolysis lignin	1.5	-	[24]

* With the addition of *o*-phenanthroline; ** wood was treated by 0.012 M HCl in 50% aqueous ethanol; *** yield is relative to the mass of the whole substrate.

**Table 2:** Comparison of the aromatic aldehydes yield in oxidation of various lignins by molecular oxygen and nitrobenzene in alkaline media.

Lignin Type	Oxidant, Catalyst, Other Remarks	Yield, wt. % of Lignin	Reference No.
Spruce wood (28% of lignin)	Air, no catalyst	11.4	[46]
Spruce wood	Air, Cu(OH)_2_ 10% of wood weight	18.9	[46]
Spruce wood	Air, MnO_2_ 10% of wood weight	18.2	[46]
Spruce wood	Nitrobenzene	20–27	Table 1
Pine wood	Oxygen, no catalyst	12.9	[42]
Pine wood	Oxygen, Cu(OH)_2_	23.1	[42]
Brown rotted Pine wood	Oxygen, Cu(OH)_2_	19.8	[42]
Aspen wood	Oxygen, CuO, flow reactor	36 *	[50]
Aspen wood	Nitrobenzene	43.6 *	Table 1
Birch wood	Oxygen, Cu(OH)_2_	43 *	[51]
Birch wood	Nitrobenzene	47 *	Table 1
Softwood lignosulfonates	Air, no catalyst	5–7	[1,45]
Softwood lignosulfonates	Oxygen, Cu(OH)_2_	12	[43]
Softwood lignosulfonates	Nitrobenzene	16	Table 1
Softwood lignosulfonates	Oxygen, Co(OH)_3_, Mn_3_O_4_	10–15.5	[1,24]
Eucalyptus lignosulfonates	Oxygen, Cu(OH)_2_	13.4 *	[52]
Kraft lignin	Oxygen, no catalyst	4.5–10.8	[49,53,54]
Kraft lignin	Nitrobenzene	13.1	Table 1
Poplar precipitated hydrolysis lignin	Oxygen, Cu(OH)_2_, Fe(OH)_3_	15 *	55

* Combined yield of vanillin and syringaldehyde.

**Table 3:** The influence of temperature on yield of vanillin and syringaldehyde from the oxidation of aspen wood by oxygen in alkaline media (see references [68,72]).

Temperature, °C	Yields, wt. % Based on Lignin		
	Vanillin	Syringaldehyde	Total Yields
170	5.5	11.5	17.0
180	7.4	15.6	23.0
190	7.8	23.4	31.2
200	7.8	19.7	27.5

**Table 4:** The influence of catalysts and pH (measured at 20 °C) on initial oxygen consumption rate; and the parameters corresponding to peak vanillin concentrations. Oxidation of lignosulfonates. Unless otherwise noted, the content of the dry sulfite liquor components in reaction solutions 180 g/L, oxygen partial pressure 0.2 MPa, reaction solution volume 60 mL (see reference [43]).

No.	Catalyst, g/L	Base, g/L	T, °C	Initial pH	Initial O_2_ Consumption Rate, g/min	Peak Vanillin Concentration Parameters		
						[V] *, g/L	Time, min	Attained pH
1	-	NaOH, 120	160	>13	0.094	6.5	>60	9.7
2	Cu(OH)_2_, 3.24	NaOH, 120	160	>13	0.110	8.7	>60	10.4
3	Cu(OH)_2_, 6.51	NaOH, 120	160	>13	0.136	11.3	40	9.8
4	Cu(OH)_2_, 9.75	NaOH, 120	160	>13	0.080	13.9	40	9.9
5	Cu(OH)_2_, 16.3	NaOH, 120	160	>13	0.103	14.7	40	10.9
6	Cu(OH)_2_, 26.0	NaOH, 120	160	>13	0.137	11.4	20	10.6
7	Cu(OH)_2_, 9.75	NaOH, 80	160	>13	0.115	10.7	20	11.0
8 **	Cu(OH)_2_, 9.75	NaOH, 40 + K_2_CO_3_, 300	160	10.8	0.056	3.3	25	10.5
9 **	Cu(OH)_2_, 9.75	K_2_CO_3_, 300	160	10.3	0.071	0.9	20	10.0
10	-	NaOH, 120	110	>13	0.018	5.4	70	-
11	Cu(OH)_2_, 9.75	NaOH, 120	110	>13	0.050	7.7	70	9.9
12	AgOH, 12.5	NaOH, 120	110	>13	0.042	5.7	70	9.5
13	Cu(OH)_2_, 9.75 + AgOH, 12.5	NaOH, 120	110	>13	0.092	7.0	>90	10.0

* Vanillin concentration; ** content of the dry sulfite liquor components in reaction solution 120 g/L.

**Table 5:** Market figures for the products of birch wood processing.

Substance	Price, 10^3^ USD/t	Global Production, 10^3^ t/year	Yield from One Tonne of Wood, kg	Value of the Product from One Tonne of Wood, USD
Vanillin	10–15	15–20	20	200–300
3,4,5-trimethoxybenzaldehyde from syringaldehyde	25–30	10–20	50	1250–1500
Levulinic acid	3	2–5	100–120	300–360
Furfural from pentosanes	1–2	90	100–120	100–240
Xylitol from xylose (in pentosanes)	6–8	20–30	130–180	800–1200

## Abbreviations

PPU	Phenylpropane units
NBO	Nitrobenzene oxidation
